# Molecularly Imprinted Polymer as Selective Sorbent for the Extraction of Zearalenone in Edible Vegetable Oils

**DOI:** 10.3390/foods9101439

**Published:** 2020-10-11

**Authors:** Paolo Lucci, Stefano David, Chiara Conchione, Andrea Milani, Sabrina Moret, Deborah Pacetti, Lanfranco Conte

**Affiliations:** 1 Department of Agri-Food, Animal and Environmental Sciences, University of Udine, via Sondrio 2/a, 33100 Udine, Italy; david.stefano@spes.uniud.it (S.D.); chiara.conchione@uniud.it (C.C.); milani.andrea.1@spes.uniud.it (A.M.); sabrina.moret@uniud.it (S.M.); lanfranco.conte@uniud.it (L.C.); 2 Department of Agricultural, Food, and Environmental Sciences, Marche Polytechnic University, Via Brecce Bianche, 60131 Ancona, Italy; d.pacetti@staff.univpm.it

**Keywords:** chromatography, maize oil, mycotoxins, zearalenone, solid-phase extraction, vegetable oils, molecularly imprinted polymers, contaminants, food safety

## Abstract

A method based on the selective extraction of zearalenone (ZON) from edible vegetable oils using molecularly imprinted polymer (MIP) has been developed and validated. Ultra-high-pressure liquid chromatography coupled with a fluorescence detection system was employed for the detection of zearalenone. The method was applied to the analysis of zearalenone in maize oil samples spiked at four concentration levels within the maximum permitted amount specified by the European Commission Regulation (EC) No. 1126/2007. As a result, the proposed methodology provided high recoveries (>72%) with good linearity (*R*^2^ > 0.999) in the range of 10–2000 μg/kg and a repeatability relative standard deviation below 1.8%. These findings meet the analytical performance criteria specified by the European Commission Regulation No. 401/2006 and reveal that the proposed methodology can be successfully applied for monitoring zearalenone at trace levels in different edible vegetable oils. A comparison of MIP behavior with the ones of QuEChERS and liquid–liquid extraction was also performed, showing higher extraction rates and precision of MIP. Finally, the evolution of ZON contamination during the maize oil refining process was also investigated, demonstrating how the process is unable to completely remove (60%) ZON from oil samples.

## 1. Introduction

Fungi are ubiquitous organisms that represent a significant problem worldwide for the cereal production sector; their presence might result in deterioration of raw materials as well as reduction of nutritional value and safety, especially if considering that some fungal species may produce secondary metabolites (such as mycotoxins) that can be harmful to human and animal health [1].

Public concern associated with food and feed contamination with toxigenic fungi or their metabolites has significantly risen during the last decades. More than 300 mycotoxins produced by hundreds of fungi species are currently known [1,2]; several of these compounds have been proven to potentially have carcinogenic, teratogenic, nephrotoxic, immunotoxic, or hemorrhagic properties. Consequently, mycotoxin contamination inevitably poses a serious threat to global food safety and leads to enormous yield and economic losses [3,4,5]. The most commonly identified mycotoxins in food and feed comprise aflatoxins, ochratoxins, trichothecenes, zearalenone, fumonisins (B1 and B2), patulin, and citrinin, among others [6].

Zearalenone (ZON) is produced by various *Fusarium* fungi, which more frequently colonize several grains under cool, wet, and humid conditions [7]. ZON exhibits low acute toxicity after oral administration in mice, rats, and guinea pigs, although it has also been shown to have estrogenic effects at relatively low levels, including infertility, reduced incidence of pregnancy, and change in progesterone levels in several animal species [8]. Moreover, zearalenone also showed other adverse health effects, such as hepato-, haemato-, and genotoxicity [9,10,11]. Fungi producing ZON mainly contaminate maize, as well as barley, oat, wheat, sorghum, millet, and rice, which therefore represent the main sources of dietary exposure to ZON. In addition, the toxin has been identified in processed cereal products like flour, malt, soybeans, and beer [12]. For that reason, several countries have set ZON tolerance levels in grains and grain products [1,13].

In 2008, Schollenberger et al. [14] reported the occurrence of zearalenone in vegetable oils. Previously, Kappenstein et al. [15] analyzed 77 oil samples, including maize, soybean, and wheat germ oils, among others. As a result, all maize oil samples were positive for ZON with a medium concentration of 170 μg/kg, while the majority of the analyzed soybean oil samples showed some ZON contamination, thought to a lesser extent. In 2011, the European Food Safety Authority (EFSA) published a scientific opinion on the risks to human health related to the occurrence of zearalenone in several foods, including vegetable oils [16]. To protect consumers from health risks, EU legislation sets maximum levels of ZON for several foodstuffs, with a ZON limit of 400 μg/kg for refined maize oil [17].

Analytical determination of ZON is usually conducted by means of high-performance liquid chromatography coupled with a selective fluorescence detector [18,19], and less frequently with UV [20]. Mass spectrometry has also been extensively applied for the simultaneous determination of several mycotoxins, including ZON [21]. In all cases, ZON analysis requires a purification step to remove matrix interferences as much as possible and to pre-concentrate the analyte to reach the required chromatographic sensitivity [22]. Liquid–liquid extraction and solid-phase extraction utilizing C18 phases [20,23] are the most used procedures reported in the literature for ZON extractions in a wide range of matrices. More recently, QuEChERS has also been reported as a valuable alternative because of its simplicity and suitability for multi-residue determination [24]. However, all these techniques may have a reduced selectivity towards zearalenone, resulting in the need for more selective and expensive detectors, such as tandem mass spectrometry. In this context, molecularly imprinted polymers (MIPs) have recently emerged as a promising and selective sorbent for the clean-up and pre-concentration of several target compounds present at low levels in food, biological, pharmaceutical, and environmental samples [25].

MIP sorbents are synthetic materials with a three-dimensional cavity that are able to selectively bind a target compound or a class of structural analogues [26]. Such specific recognition sites are created by polymerizing functional monomers and cross-linkers in the presence of a template molecule. Then, the size, shape, and binding group orientation of the recognition sites derived from the extraction of the template allow the cavities to specifically rebind the target compound, mimicking the very selective interaction between antibodies and antigens [27,28,29].

In addition, imprinted polymers are more stable compared to biological receptors because of their greater tolerance to elevated temperature and pressure, as well as their stability toward extreme pH ranges and organic solvents. Another benefit is represented by the fact that their synthesis is also reasonably easy and cheap with respect to the purification process of natural antibodies [30,31].

MIP technology has recently found application for the analysis of zearalenone in grains, beer, and corn flakes, among others [32,33,34]. However, the usefulness of MIP for selective ZON extraction in oil samples has not been yet investigated. Therefore, the aim of the present research was to develop and validate a reliable method for the extraction of ZON from edible vegetable oils using a molecularly imprinted polymer (AFFINIMIP^®^SPE Zearalenone) as a solid-phase extraction sorbent (MIP-SPE). A comparison with other extraction procedures, such as QuEChERS and liquid–liquid approaches, was also performed. The optimized and validated method was then applied to examine the occurrence of ZON in different types of vegetable oils purchased at local markets. A supplementary goal was to investigate the concentration and reduction levels of ZON in crude maize oil submitted to the refining process.

## 2. Materials and Methods

### 2.1. Chemicals and Reagents

All chemicals and reagents employed in this work were obtained from Sigma-Aldrich Chemical Co. Ltd. (Milan, Italy). Methanol, diethyl ether, acetic acid, and hexane were of analytical grade, while acetonitrile (ACN) was of HPLC grade. Water was purified with a Milli-Q system (Millipore Corporation, Bedford, MA, USA). The *d*-solid-phase extraction (SPE) sorbent C18 and magnesium sulphate (MgSO_4_) anhydrous were also from Sigma-Aldrich (Milan, Italy). Zearalenone standard solution (100 μg/mL in ACN) was purchased from Affinisep (Petit-Couronne, France). Working standard solutions were prepared weekly using ACN as the diluting solvent. All solutions were kept in the refrigerator at 4 °C and then used within one month. Molecularly imprinted polymers (AFFINIMIP^®^ SPE Zearalenone) were from Affinisep (Petit-Couronne, France). Non-imprinted polymer (NIP), synthetized using the same procedure of the MIP, but in the absence of the template, was also provided by Affinisep (Petit-Couronne, France).

### 2.2. Oil Samples

Eleven commercial edible oil samples, namely maize oil (*n* = 7), soybean oil (*n* = 3), and rice oil (*n* = 1), were purchased at local markets. Maize oil samples collected at different steps of the refining procedure (unrefined, neutralized, bleached, and refined oils) were also obtained from an Italian oil refinery. Oil refining conditions have already been described elsewhere [35].

The samples were stored in the dark at –18 °C until chemical analyses were conducted.

### 2.3. Extraction and Clean-Up Using MIP-SPE

Empty 3 mL SPE cartridges were packed with 100 mg of imprinted and non-imprinted polymer particles between two fritted polypropylene disks. A preliminary conditioning step using 3 mL of diethyl ether was performed on both MIP- and NIP-SPE columns before each usage.

For the method validation, maize oil (2 g) was fortified with specific amounts of ZON (from 10 to 2000 µg/kg) and diluted with 6 mL of diethyl ether to obtain the loading solution. A total of 3 mL of this loading solution (corresponding to 1 g of maize oil) were loaded onto the MIP-SPE column with a percolating flow rate of no more than 2–3 drops per second. Then, 6 mL of diethyl ether was added to the polymer. Residual solvent was removed by applying full vacuum for 2 min, and the column was washed with 6 mL of water/acetic acid/acetonitrile (58:2:40, *v*/*v*/*v*). Zearalenone was finally eluted with 4 mL of 2% acetic acid methanol solution. Extraction recovery was determined by comparing the amount of ZON extracted by MIP-SPE with the theoretical amount added to the sample before extraction and corresponding to 100%. The matrix effect was estimated by comparing the slopes of standard curves in pure solvent and the matrix-matched calibration curve at the ZON concentration range used for MIP-SPE validation (20–300 µg/kg). The matrix effect was calculated as: 100 × (1 − (solvent slope)/(matrix slope)) [36].

### 2.4. Liquid–Liquid Extraction Procedure

Liquid–liquid extraction (LLE) of ZON was conducted using the methodology reported elsewhere [14]. Briefly, an aliquot of oil sample (5 g) was extracted by 50 mL of hexane and 50 mL of acetonitrile/water (75:25, *v*/*v*). The mixture was shaken and centrifuged at 3400 rpm at room temperature for 10 min. Then, the lower phase was collected and placed into a separatory funnel, and 50 mL of hexane were added. Once a complete separation of the two phases was achieved, solvent from the lower part was removed with a rotary evaporator, and the residue was suspended in 0.5 mL of ACN before UHPLC analysis.

### 2.5. QuEChERS Extraction Procedure

The QuEChERS procedure was carried out using the protocol reported by Sharmili et al. [37], with slight modifications. A total of 3 g of oil sample was weighed into a 50 mL centrifuge tube, followed by the addition of 10 mL of ACN. The mixture was shaken at 180 rpm for 10 min and then centrifuged at 3700 rpm for 2 min. An aliquot of the upper ACN layer (5 mL) was transferred to a second centrifuge tube containing 200 mg *d*-SPE sorbent (C18) and 100 mg MgSO_4_ anhydrous. After shaking manually, the extract was centrifuged at 3700 rpm for 2 min. A total of 3 mL of supernatant was collected into a clean glass tube, and the solvent was removed at 40 °C with a gentle stream of nitrogen. The residue was dissolved in 0.5 mL of ACN before analysis.

### 2.6. HPLC-FLD Analysis

The HPLC system used for the MIP-SPE, LLE, and QuEChERS extracts’ evaluation consisted of a Shimadzu UHPLC Nexera (Shimadzu, Kyoto, Japan) equipped with an RF-20Axs fluorimetric detector. For fluorescence acquisition, 275 and 450 nm were chosen as the excitation and emission wavelengths, respectively. The analytical column was an Agilent Poroshell 120 EC-C18 (4.6 × 150 mm, 2.7 μm particle size) thermostated at 30 °C. The Shimadzu LabSolutions LC-MS was used for data acquisition and analysis.

The chromatographic separation of oil extracts was performed using a gradient elution with water containing 2% acetic acid (solvent A) and ACN (solvent B) as the mobile phase components at a flow rate of 450 μL/min. The following gradient was applied: 0–0.5 min, isocratic step at 50% B; 0.5–9 min, linear gradient from 50 to 80% B; 9–11 min, linear gradient from 80 to 100% B; 11–13 min, isocratic elution at 100% B; 13.01–18 min, back to initial conditions at 50% of solvent B. Injection volumes for each sample were 2 μL.

## 3. Results and Discussion

### 3.1. Analytical Parameters of the HPLC Method

The quantitative analysis of ZON was conducted using an external standard calibration curve. For this purpose, a curve was constructed by injecting different amounts of ZON standard (0.05–12.5 ng on column). A good correlation coefficient (*R*^2^) of 0.999 was obtained. The precision of the UHPLC method was determined by evaluating the repeatability obtained by injecting nine times with two different quantities (2 and 0.2 ng on column) of ZON standard. The results were satisfactory, with a repeatability relative standard deviation (RSDr) ≤ 1.6% in all cases. The proposed methodology also demonstrates sufficient sensitivity for the determination of ZON at ppb levels: The instrumental limit of detection (ILOD) was 10 pg injected on column (calculated at signal-to-noise ratio of 3:1), while the limit of quantification (ILOQ) was 32 pg injected on column (calculated at signal-to-noise ratio of 10:1).

### 3.2. Evaluation of MIP-SPE Procedure for the Selective Extraction of Zearalenone

**Figure 1:**
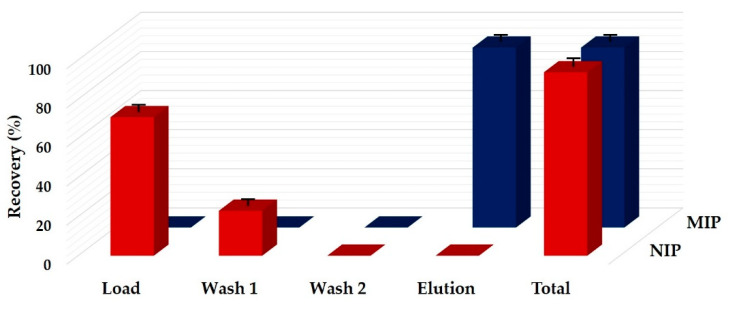
Recovery profile of zearalenone obtained on the non-imprinted polymer (NIP) and molecularly imprinted polymer (MIP) using pure solvent. Load: 3 mL of diethyl ether spiked with zearalenone at the level of 125 ng/mL; Wash 1: 6 mL of diethyl ether; Wash 2: 6 mL of water/acetic acid/acetonitrile (58:2:40 *v*/*v*/*v*); Elution: 4 mL of methanol/acetic acid (98:2 *v*/*v*).

To assess the potential of MIP-SPE for the selective extraction of ZON, recovery experiments were firstly conducted using pure solvent in the absence of matrix interferents. For this purpose, once the polymer was properly conditioned, 3 mL of diethyl ether spiked with 125 ng/mL of zearalenone were loaded onto the MIP column. The same experiment was also carried out on NIP to highlight the involvement of the MIP active sites on the selective recognition of ZON. Under organic conditions, zearalenone was completely retained by the MIP, while a significant quantity of the mycotoxin was stripped off the NIP during the loading step. Then, as shown in Figure 1, a complete desorption of zearalenone was observed in the non-imprinted polymer during the first washing step consisting of 6 mL of diethyl ether, while the analyte was completely retained on the MIP cartridge. This is probably due to the absence on NIP of the imprinted cavities characterized by functional groups with the same spatial arrangement of the target compound and that are responsible for the higher selectivity and strength of the MIP’s binding ability.

**Figure 2:**
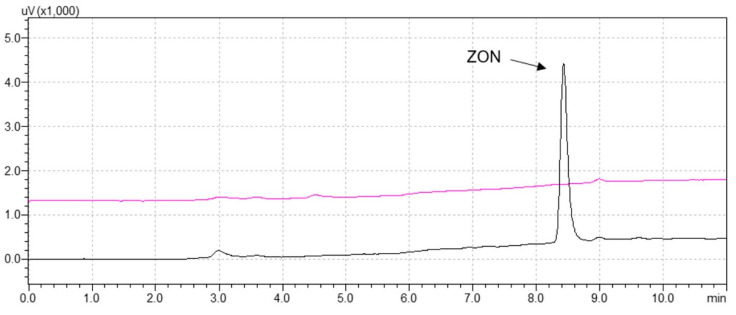
Chromatograms of elution fractions obtained by loading 3 mL of diethyl ether spiked at level of 125 ng/mL after the NIP- (pink trace) and MIP-solid-phase extraction (SPE) (black trace) clean-up procedures; ZON = zearalenone.

Then, the polymers were dried under vacuum, and 6 mL of water/acetic acid/acetonitrile (58:2:40 *v*/*v*/*v*) were loaded. This second washing step is crucial, especially when analyzing real samples. In fact, the use of a more polar solvent should be capable of disrupting non-specific interactions between the polymer and medium-polar matrix compounds. Once again, no release of ZON was observed from the MIP. Finally, zearalenone was eluted from the MIP-SPE cartridge with 4 mL of methanol/acetic acid (98:2 *v*/*v*). A comparison of the UHPLC traces acquired by analyzing elution fractions obtained from the MIP and NIP cartridges is reported in Figure 2. The NIP chromatogram reveals the lack of ZON retention on the non-imprinted polymer and, therefore, the effectiveness of the washing procedure in the enhancement of MIP selectivity by disrupting non-specific interactions. On the contrary, the results achieved from the analysis of the imprinted polymer elution fraction indicated high extraction recoveries (93–95%).

Then, in order to prove the ability of the MIP for the extraction of ZON in real samples, a maize oil sample that was previously analyzed to confirm the absence of the mycotoxin was fortified at a level of 150 μg/kg and submitted to the MIP-SPE extraction procedure.

**Figure 3:**
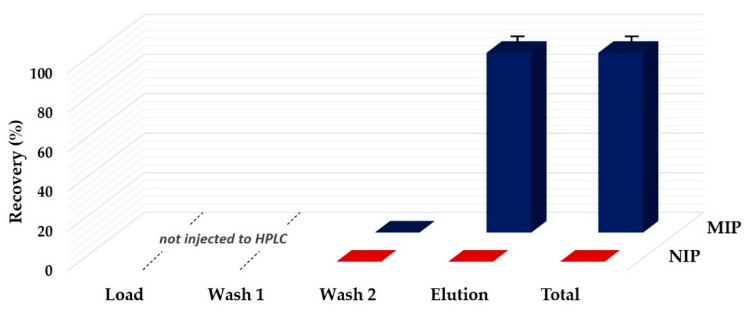
Recovery profile of zearalenone (ZON) obtained on NIP and MIP using a maize oil sample spiked with ZON at a level of 150 μg/kg. Load: 3 mL of maize oil/diethyl ether (1:3 *v*/*v*); Wash 1: 6 mL of diethyl ether; Wash 2: 6 mL of water/acetic acid/acetonitrile (58:2:40 *v*/*v*/*v*); Elution: 4 mL of methanol/acetic acid (98:2 *v*/*v*).

In this experiment, only fractions obtained from the second washing step and the elution step were collected and analyzed by UHPLC, since the previous fractions (loading and Wash 1) could have contaminated the analytical column because of their high content of non-polar fatty compounds, mainly triacylglycerols. As shown in Figure 3, the results from MIP elution fractions confirmed high extraction efficiencies (91–93%), while the total amount of ZON was lost during the loading and/or the first washing steps on the NIP.

These findings suggest that ZON was also efficiently rebound by the MIP when analyzing real complex samples, such as maize oil, without suffering from matrix interferences.

### 3.3. Validation of the MIP-SPE Procedure

The accuracy of the proposed MIP-SPE method was assessed by means of recovery experiments by analyzing maize oil samples spiked at four different concentration levels within the maximum ZON content allowed by the European Commission Regulation (EC) No. 1126/2007 [17]. As mentioned before, regarding refined maize oil, a maximum allowable level of 400 µg/kg has been fixed. Therefore, recoveries were determined by evaluating the extraction ability of MIP-SPE in maize oil samples at levels of 20, 100, 200, and 300 µg/kg of ZON. All experiments were done in triplicate. The thusly obtained data are given in Table 1.

The average recovery for samples spiked in the range of 100–300 μg/kg varied from 89 to 93%. Lower but still satisfactory recoveries (71–74%) were also obtained when analyzing refined maize samples spiked at the lowest selected level (20 μg/kg of ZON), thus indicating the satisfactory suitability of the proposed methodology. In fact, the European Commission Regulation (EC) No. 401/2006 [38] establishes ZON recoveries between 70–120% for concentration above 50 µg/kg, and from 60 to 120% for ZON levels below 50 µg/kg. In addition, the procedure also allows for precise and accurate ZON determination, as demonstrated by the repeatability standard deviation (sr) between 1.3 and 1.7 µg/kg and repeatability relative standard deviation (RSDr) between 1.4 and 1.8%. Once again, these findings confirm how the herein-proposed MIP-SPE method meets the Commission Regulation 401/2006 performance criteria (RSDr ≤ 25% for ZON level above 50 μg/kg; RSDr ≤ 40% for ZON level below 50 μg/kg). The matrix effect was –1.3%, thus indicating that the MIP-SPE procedure also exhibits negligible relative matrix effects. The linearity of the method, which was assessed by analyzing a series of maize oil samples fortified over a concentration range equivalent to 10–2000 µg/kg, provided a very good linear relationship, with *R*^2^ higher than 0.998. With regard to the analytical sensitivity, the method limit of detection (MLOD) was determined to be 1.2 µg/kg, while the method limit of quantification (MLOQ) was 4 µg/kg, based on signal-to-noise ratios of 3:1 and 10:1, respectively.

After all, a comparison between the performances of MIP-SPE, LLE, and QuEChERS procedures was also conducted using a refined maize oil sample spiked at 200 µg/kg.

**Figure 4:**
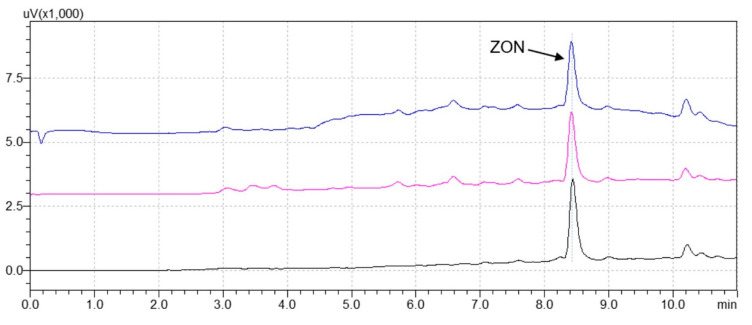
Chromatograms of a refined maize oil sample spiked with 200 μg/kg of ZON after extract clean-up with (blue trace) QuEChERS, (pink trace) liquid–liquid extraction (LLE), and (black trace) MIP-SPE.

A comparison of UHPLC traces obtained after applying the different methodologies is shown in Figure 4. All methods provide acceptable chromatograms for the quantification of ZON, even if a slight baseline drift can be observed in the LLE and QuEChERS traces because of some co-extracted matrix interferences.

On the other hand, as reported in Table 2, MIP extraction recoveries were higher compared to LLE and QuEChERS, where extraction efficiencies ranged from 58 to 70%. In addition, contrarily to MIP-SPE, both the LLE and QuEChERS procedures failed to fulfill the European Commission Regulation (EC) No. 401/2006 requirement of recovery rates (>70% for ZON level above 50 µg/kg).

As result, the MIP-SPE demonstrated higher extraction efficiency and precision, as well as its suitability as a sample preparation technique for the monitoring of ZON in oil samples. This was also confirmed by the analysis of a more complex matrix, such as crude maize oil, where impurities, pigments, free fatty acids, and oxidation products, as well as residual proteins, phosphatides, and a part of the undesirable flavors, colors, and odors, have not been removed by the refining process. For this experiment, a blank crude unrefined maize oil sample was spiked at 200 μg/kg of ZON before being submitted to the MIP-SPE, LLE, and QuEChERS extraction methods.

**Figure 5:**
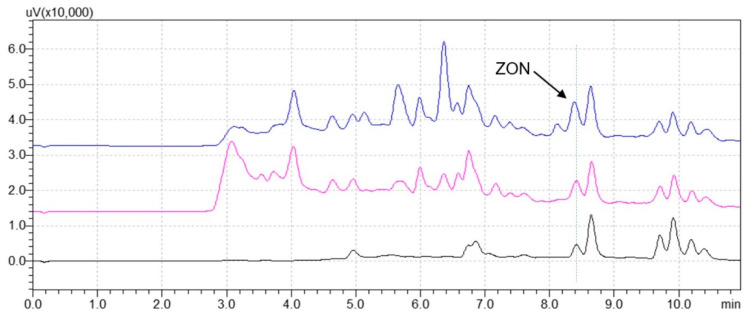
Chromatograms of crude maize oil spiked with 200 μg/kg of ZON after extract clean-up with QuEChERS (blue trace), LLE (pink trace), and MIP-SPE (black trace).

As can be seen in Figure 5, a cleaner chromatogram can be obtained using MIP-SPE when compared to the ones achieved with LLE and QuEChERS. In fact, the MIP-SPE chromatogram shows the absence of interferent peaks overlapping with ZON, which allows the proper quantification of the mycotoxin with an average recovery of 73%. On the contrary, LLE and QuEChERS lead to recovery overestimations (131 and 267%, respectively) because of the presence of co-extracted matrix interferents that are coeluting with ZON.

### 3.4. Application of MIP-SPE in Commercial Edible Oils

A total of 11 vegetable oil samples, including maize oil (*n* = 7), soybean oil (*n* = 3), and rice oil (*n* = 1), were randomly purchased at local markets and submitted to the MIP-SPE procedure in order to evaluate the occurrence levels of zearalenone and to prove the applicability of the method.

As can be seen in Table 3, we found that 8 out of the 11 samples were contaminated with zearalenone. However, samples exceeding legally fixed maximum levels were not found. In the maize oil samples, a maximum content of 51.1 µg/kg was detected, with an average value of 26.2 µg/kg. On the contrary, only in one of the three soybean oil samples was ZON found, at a level of 12.7 µg/kg. Finally, the amount of zearalenone in the rice oil was lower when compared to the other selected samples.

Two previous publications on the occurrence of ZON in maize oils reported medium levels of 170 µg/kg (analyzed samples: 38, contaminated ones: 38) and 505 µg/kg (analyzed samples: 17, contaminated ones: 9) [14,15]. Hence, on the basis of previous studies, the average ZON level found in our research is comparatively lower, and in contrast to the previous reports, a violation of the current EU legal limit (400 µg/kg) for ZON was not noticed. This may be partly due to a greater awareness and concern amongst producers about zearalenone contamination in maize oil following the introduction of the EU legal limit in 2006 [38]. Regarding soybean oil, Kappenstein et al. [15] found that 70% of the selected soybean oil samples (*n* = 20) were contaminated with a medium ZON level of 4 μg/kg, while Schollenberger et al. [14] detected ZON in three out of 14 analyzed soybean oils with average and maximum concentrations of 24 and 46 μg/kg, respectively.

Rice oil was previously investigated by Siegel et al. [39], but zearalenone was not found. Overall, the ZON content found in our soybean and rice oil samples was lower compared to the one observed in maize oil, as expected. Nevertheless, to date, there is still not an EU legal limit for zearalenone in soybean and rice oils.

### 3.5. Application of MIP-SPE in Maize Oil Samples at Various Stages of the Refining Process

Another goal of this work was to investigate the loss of ZON in maize oil during the refining process. To do this, a crude maize oil naturally contaminated with ZON (73.9 μg/kg) was selected and then monitored by collecting oil sample fractions at different steps of the refining process (crude oil, neutralized oil, bleached oil, and refined oil).

With respect to the ZON evolution during refining, a significant decrease of the mycotoxin occurred during the process (Table 4). In particular, after the neutralization, about the 29% of ZON was lost, while, following the bleaching, ZON seemed to be increased; this could be interpreted as a concentration effect because of the removal of oil mass at this stage, which does not necessarily involve ZON. After the last step (deodorization), 60% of the initial concentration was lost. Therefore, at the end of refining process, about 40% of the initial level of ZON remained in the final refined oil sample. A different trend was previously found by Kamimura et al. [40], who reported a complete removal of deoxynivalenol, nivalenol, and ZON in refined crude corn germ oil. However, this disagreement may be attributable to a difference in the conditions employed for the refining procedure, as well as in the sensitivity of the analytical procedure used for multi-mycotoxin detection. In fact, our results are in accordance with earlier data reporting the presence of ZON in both crude and refined soybean, sunflower, and corn germ oils, though, to a lesser extent, in the refined ones [14]. On the whole, our findings corroborate that ZON is not completely removed by the refining treatment. Hence, the assessment of the initial levels of zearalenone contamination becomes increasingly important for the oil industry, and, in view of this, the MIP-SPE technique could be a valuable analytical option.

## 4. Conclusions

Mycotoxins indubitably represent one of the most important contaminants in food matrices, and a great effort has been expended in the recent past in the development of analytical solutions able to guarantee fast and reliable measurements of such contaminants in different types of food samples. Nowadays, the trend of mycotoxin detection in food matrices is the use of single methods capable of analyzing multi-residue mycotoxins, with a significant improvement in laboratories’ throughput. This is now possible not only because of the recent advances in separation sciences, but also because of the developments of fast and accurate mass spectrometry analyzers. However, multi-residue methods usually require sophisticated and expensive instruments as well as highly trained personnel. For this reason, it is still important to continue to try to develop simpler and cheaper methods that can also allow small laboratories or industries to be able to carry out targeted and accurate monitoring of some specific contaminants. In this context, the present study presented a simple but selective and reliable MIP-SPE/UHPLC-FLD method for the quantification of zearalenone in edible vegetable oils. Moreover, to the best of our knowledge, this is the first report on the use of MIP as a selective sorbent for solid-phase extraction of ZON in oil samples. The method, which does not require the use of expensive instrumentation, has been developed and validated, fulfilling the performance criteria laid down in the European Commission Regulation (EC) No. 401/2006 [38]. The herein-proposed analytical procedure was successfully applied to the analysis of different types of vegetable oils purchased at local markets as well as for the assessment of the ZON evolution during the refining process of maize oil. For these reasons, the developed MIP-SPE method may represent a valuable alternative to more sophisticated analytical approaches, and it is considered to be well suited for the monitoring of the current EU legal limit for zearalenone in refined maize oil as well as for the analysis of other vegetable oils, contributing to improving food safety and consumer protection.

## Figures and Tables

**Table 1:** Recovery of zearalenone (ZON) from spiked maize oil samples.

Spike (µg/kg)	Recovery (%)			Main (%)	sr (µg/kg)	RSDr (%)
	a	b	c			
20	71	74	71	72	1.3	1.8
100	95	94	92	93	1.3	1.4
200	91	87	88	89	1.4	1.6
300	93	90	90	91	1.7	1.8

*n* = 3 (a,b,c); sr = repeatability standard deviation; RSDr = repeatability relative standard deviation.

**Table 2:** Recovery of ZON from spiked maize oil samples using different extraction procedures.

Procedure	Recovery (%)			Main (%)	sr (µg/kg)	RSDr (%)
	a	b	c			
MIP-SPE	91	87	88	89	1.4	1.6
LLE	68	69	58	65	4.9	7.5
QuEChERS	70	65	62	65	3.2	5.1

*n* = 3 (a,b,c); sr = repeatability standard deviation; RSDr = repeatability relative standard deviation.

**Table 3:** Zearalenone content (µg/kg) of commercial oil samples purchased at local markets.

Type of Oil	Sample	Level of ZON (µg/kg)
Maize oil	1	12.5 ± 1
	2	27.1 ± 2
	3	22.6 ± 1
	4	20.1 ± 1
	5	-
	6	51.1 ± 2
	7	23.8 ± 1
Soybean oil	1	-
	2	12.7 ± 0
	3	-
Rice oil	1	5.7 ± 0

**Table 4:** Effect of the refining stages on zearalenone content (mg/kg oil) of maize oil.

Sample	Level of ZON (µg/kg)
Crude	73.9 ± 3
Neutralized	52.8 ± 1
Bleached	57.7 ± 2
Refined	29.3 ± 2
